# Supplementary material for: Bovine Lactoferrin and Hen Ovotransferrin Affect Virulence Factors of Acute Hepatopancreatic Necrosis Disease (AHPND)-Inducing Vibrio parahaemolyticus Strains
Source: Microorganisms. 2023 Dec 2;11(12):2912. doi: 10.3390/microorganisms11122912 (PMC10745944; doi:10.3390/microorganisms11122912)
Supplement: Supplementary file 1 [file microorganisms-11-02912-s001.zip › microorganisms-2712850-supplementary.pdf]

Supplementary Material

Bovine Lactoferrin and Hen Ovotransferrin Affect Virulence Factors of Acute Hepatopancreatic Necrosis Disease (AHPND)-Inducing *Vibrio parahaemolyticus* Strains

Marieke Vandeputte, Margaux Verhaeghe, Lukas Willocx, Peter Bossier, Daisy Vanrompay*

* Correspondence: daisy.vanrompay@ugent.be

1 Supplementary Figures and Tables

1.1 Supplementary Figures

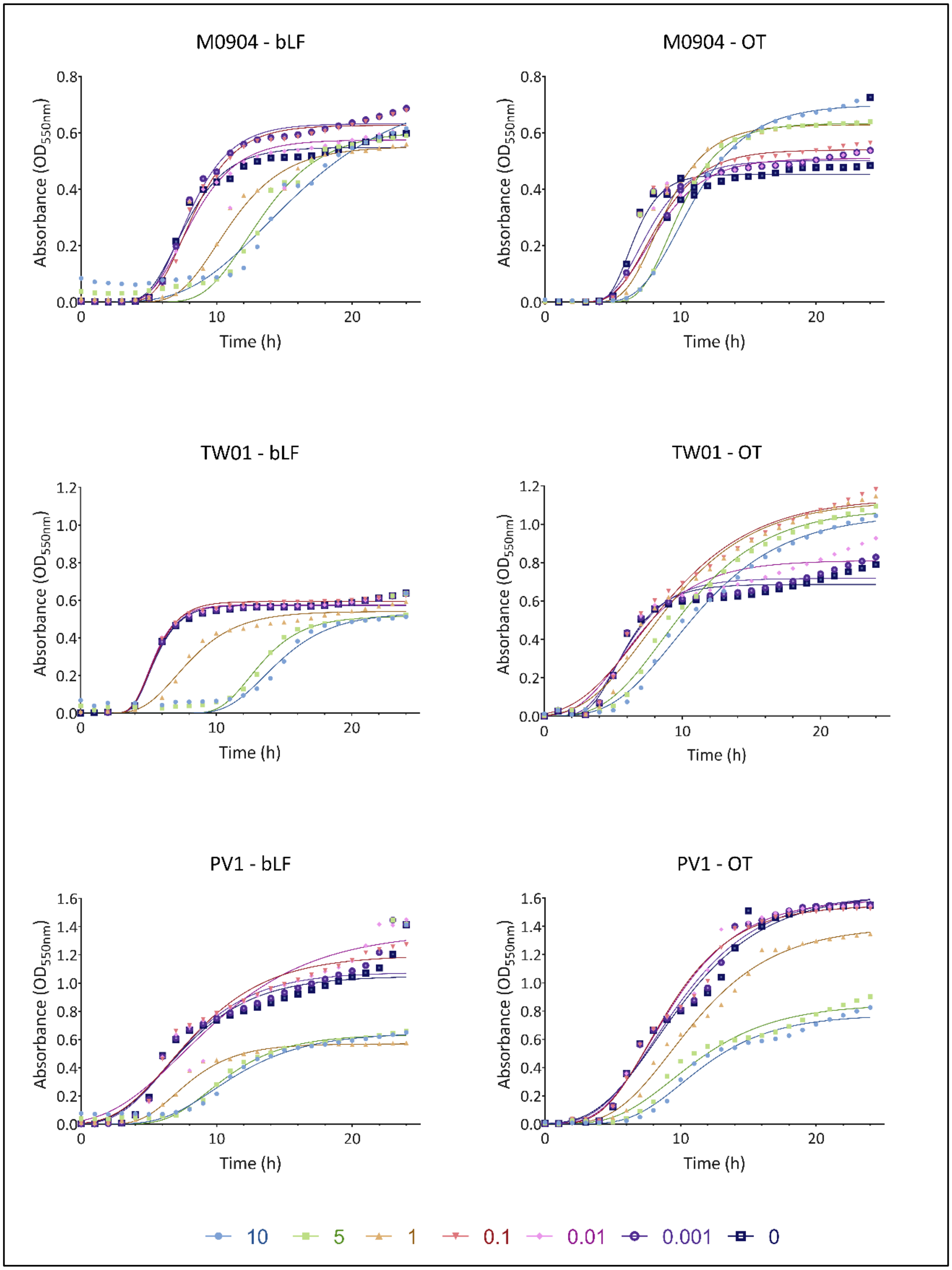

Supplementary Figure S1. Growth curves of Vp M0904, Vp TW01 and Vp PV1, with a starting concentration of 105 CFU/ml, and incubation with bLF and OT at concentrations 0, 0.001, 0.01 0.1, 1, 5 and 10 mg/ml. Individual points represent the mean OD550 (n = 3), curves are the non-linear fitting to the modified Gompertz model.

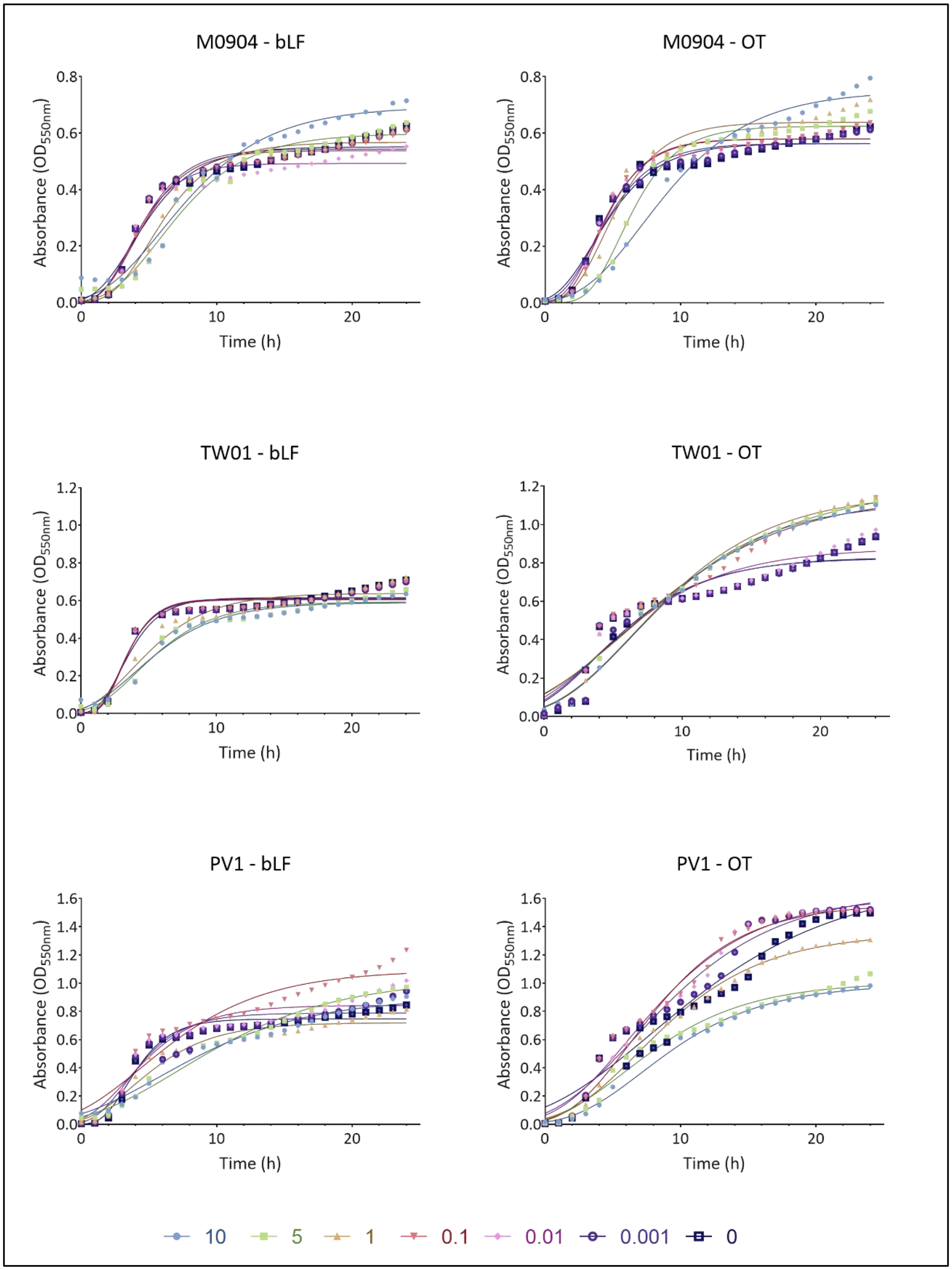

Supplementary Figure S2. Growth curves of Vp M0904, Vp TW01 and Vp PV1, with a starting concentration of 107 CFU/ml, and incubation with bLF and OT at concentrations 0, 0.001, 0.01 0.1, 1, 5 and 10 mg/ml. Individual points represent the mean OD550 (n = 3), curves are the non-linear fitting to the modified Gompertz model.

1.2 Supplementary Tables

Supplementary Table S1. A summary of the bacterial growth parameters of Vp M0904, Vp TW01 and Vp PV1 with populations 105 and 107 CFU/ml, derived from the modified Gompertz model. YM represents the maximum population, expressed as values of optical density (OD550), Lag represents the length of the lag time in h. Between brackets, significant differences between transferrin treatment and relative control (0 mg/ml) are indicated by asterisks, *p<0.05, **p<0.01, ***p<0.001, ****p<0.0001. (/: modified Gompertz model was not able to calculate growth parameters due to too long lag phase or completely flat curve).

***Vp* M0904**					
	**Treatment**	**bLF**		**OT**	
	**(mg/ml)**	**YM**	**Lag**	**YM**	**Lag**
**10^5 CFU/ml**	**10**	0.750 (**)	7.949 (***)	0.699 (****)	6<br>.945 (****)
	**5**	0.607 (ns)	9.155 (****)	0.633 (****)	7.016 (****)
	**1**	0.551 (ns)	7.215 (***)	0.628 (****)	5.887 (**)
	**0.1**	0.626 (ns)	5.431 (ns)	0.540 (****)	5.159 (ns)
	**0.01**	0.575 (ns)	5.094 (ns)	0.509 (ns)	5.050 (ns)
	**0.001**	0.632 (ns)	5.160 (ns)	0.501 (ns)	4.881 (ns)
	**0**	0.548	4.899	0.453	4.765
**10^7 CFU/ml**	**10**	0.694 (****)	1.585 (ns)	0.750 (****)	2.739 (**)
	**5**	0.599 (ns)	2.129 (ns)	0.624 (**)	3.214 (***)
	**1**	0.567 (ns)	2.284 (ns)	0.639 (***)	1.951 (ns)
	**0.1**	0.537 (ns)	1.490 (ns)	0.580 (ns)	1.698 (ns)
	**0.01**	0.492 (*)	1.411 (ns)	0.580 (ns)	1.472 (ns)
	**0.001**	0.543 (ns)	1.435 (ns)	0.564 (ns)	1.181 (ns)
	**0**	0.551	1.057	0.563	0.900
***Vp* TW01**					
	**Treatment**	**bLF**		**OT**	
	**(mg/ml)**	**YM**	**Lag**	**YM**	**Lag**
**10^5 CFU/ml**	**10**	0.534 (ns)	10.796 (****)	1.052 (****)	4.970 (*)
	**5**	0.515 (*)	10.310 (****)	1.082 (****)	4.435 (ns)
	**1**	0.540 (ns)	4.609 (ns)	1.122 (****)	3.001 (ns)
	**0.1**	0.594 (ns)	3.797 (ns)	1.138 (****)	2.337 (ns)
	**0.01**	0.575 (ns)	3.796 (ns)	0.811 (**)	2.433 (ns)
	**0.001**	0.572 (ns)	3.869 (ns)	0.718 (ns)	3.073 (ns)
	**0**	0.576	3.788	0.687	3.285
**10^7 CFU/ml**	**10**	0.589 (ns)	0.546 (ns)	1.119 (***)	0.844 (ns)
	**5**	0.593 (ns)	1.000 (ns)	1.209 (****)	-1.167 (ns)
	**1**	0.638 (ns)	0.655 (ns)	1.165 (***)	0.813 (ns)
	**0.1**	0.608 (ns)	1.461 (ns)	1.157 (***)	-1.125 (ns)
	**0.01**	0.609 (ns)	1.549 (ns)	0.877 (ns)	-1.138 (ns)
	**0.001**	0.604 (ns)	1.434 (ns)	0.831 (ns)	-0.808 (ns)
	**0**	0.615	1.274	0.826	-0.599
***Vp* PV1**					
	**Treatment**	**bLF**		**OT**	
	**(mg/ml)**	**YM**	**Lag**	**YM**	**Lag**
**10^5 CFU/ml**	**10**	0.647 (****)	5.958 (**)	0.773 (****)	6.370 (**)
	**5**	0.635 (****)	6.317 (***)	0.851 (****)	5.234 (ns)
	**1**	0.569 (****)	4.424 (ns)	1.400 (**)	5.126 (ns)
	**0.1**	1.196 (ns)	2.618 (ns)	1.550 (ns)	4.244 (ns)
	**0.01**	1.364 (***)	2.103 (ns)	1.592 (ns)	4.173 (ns)
	**0.001**	1.076 (ns)	2.738 (ns)	1.627 (ns)	3.957 (ns)
	**0**	1.050	2.533	1.633	3.835

**10^7 CFU/ml**	**10**	0.901 (*)	-0.521 (ns)	0.993 (****)	2.120 (*)
	**5**	1.033 (***)	0.587 (ns)	1.007 (****)	0.840 (ns)
	**1**	0.720 (ns)	0.469 (ns)	1.355 (**)	1.568 (ns)
	**0.1**	1.092 (****)	-0.675 (ns)	1.562 (ns)	1.775 (ns)
	**0.01**	0.842 (ns)	0.361 (ns)	1.610 (ns)	0.911 (ns)
	**0.001**	0.788 (ns)	0.940 (ns)	1.652 (ns)	0.683 (ns)
	**0**	0.747	1.547	1.731	-0.184
